# Commentary: Impact of prognostic nutritional index and geriatric nutritional risk index on prognosis in elderly patients with early-stage prostate cancer

**DOI:** 10.3389/fnut.2026.1863187

**Published:** 2026-08-10

**Authors:** Tingting Bai, Tong Liu, Peng Zhang

**Affiliations:** 1 Department of Clinical Laboratory, The First Affiliated Hospital of Dalian Medical University, Dalian, China; 2 The First Affiliated Hospital of Dalian Medical University, Dalian, China; 3 Department of Urology, The First Affiliated Hospital of Dalian Medical University, Dalian, China

**Keywords:** competing risks, geriatric nutritional risk index, geriatric oncology, prognostic nutritional index, prostate cancer

We read with great interest the recent article by Peker et al., titled “Impact of prognostic nutritional index and geriatric nutritional risk index (GNRI) on prognosis in elderly patients with early-stage prostate cancer,” published in *Frontiers in Nutrition* (1). The authors investigated the prognostic significance of the prognostic nutritional index (PNI) and the geriatric nutritional risk index (GNRI) in elderly patients with early-stage prostate cancer and reported that poorer nutritional status was associated with worse survival outcomes (1). This study is clinically meaningful because it highlights the potential role of simple, routinely available nutritional indices in risk stratification for an elderly oncologic population.

Nutritional status is an important component of prognostic assessment in geriatric oncology. In elderly patients with early-stage localized prostate cancer, host-related factors and non-cancer mortality may substantially influence overall prognosis because cancer-specific survival is often relatively prolonged (2, 3). Against this background, PNI, which incorporates serum albumin and peripheral lymphocyte count, and GNRI, which incorporates serum albumin and body-weight-related information, are attractive because they can be calculated from routine clinical data and may help identify vulnerable patients who require closer monitoring or nutritional intervention (4). The authors are to be commended for addressing an important and often underexplored dimension of oncologic prognosis, namely the contribution of nutritional and systemic physiological status in an elderly population that is frequently underrepresented in clinical research. Nevertheless, several methodological aspects warrant further consideration, as they may further clarify the interpretation, robustness, and clinical applicability of the findings.

The study population comprises elderly patients with early-stage localized prostate cancer, a clinical context in which non-cancer-related mortality is common. In older men with localized or early-stage prostate cancer, the absolute risk of death from competing causes may be substantial because prostate cancer-specific mortality is often low over intermediate-term follow-up, whereas deaths from cardiovascular disease, other malignancies, infections, and age-related comorbidities accumulate with age (3). Several population-based studies of localized prostate cancer have shown that non-cancer mortality can exceed prostate cancer-specific mortality, particularly among elderly patients and those with low-risk or early-stage disease (3, 5). The authors applied Kaplan–Meier methods and Cox proportional hazards models to evaluate overall survival (1). Although overall survival is an objective and clinically meaningful endpoint, its composite nature precludes differentiation between cancer-specific mortality and mortality related to comorbid conditions or general frailty. Consequently, distinguishing cancer-specific mortality from competing non-cancer mortality could help clarify whether the observed associations of low PNI and GNRI with poorer survival primarily reflect tumor-related processes or a broader state of systemic vulnerability.

To address this issue, the evaluation of prostate cancer specific mortality defined as death directly attributable to prostate cancer would provide more etiologically informative insight. However, analysis of this endpoint in an elderly cohort introduces an important methodological consideration. If conventional Kaplan–Meier methods are applied, non-cancer deaths are treated as censored observations. This approach violates the assumption of independent censoring and may lead to overestimation of the cumulative incidence of prostate cancer related death. The magnitude of this overestimation is expected to increase as the proportion of competing non-cancer deaths increases. Therefore, in an elderly early-stage prostate cancer cohort, even a modest number of prostate cancer-specific deaths accompanied by a larger number of competing deaths could affect the estimated probability of prostate cancer-related mortality.

In this setting, a competing risk framework is more appropriate. Methods such as the Fine–Gray subdistribution hazard model allow direct estimation of cumulative incidence and provide a more accurate assessment of prognostic effects in the presence of competing events. Contemporary methodological guidance also recommends using cumulative incidence functions rather than the complement of the Kaplan–Meier estimator when competing events are present, and emphasizes transparent reporting of Fine–Gray analyses in studies of time-to-event outcomes (3). If cause of death data is available, supplementary analysis using a competing risk model would further enhance the clinical interpretability of the findings.

A second methodological issue concerns the inclusion of both PNI and GNRI within the same multivariable Cox regression model. These indices are partially dependent on shared biological components, particularly serum albumin, and therefore represent structurally related variables rather than fully independent predictors. PNI is commonly calculated from serum albumin and total lymphocyte count, whereas GNRI incorporates serum albumin and a body-weight-related component (4). Therefore, although the two indices are not identical, they partially overlap through albumin and both reflect related aspects of nutritional and systemic physiological status.

Although variance inflation factors were reportedly assessed, this does not fully resolve the interpretive concern arising from the simultaneous inclusion of two structurally overlapping nutritional indices. Statistical collinearity, assessed using variance inflation factors, tolerance values, condition indices, or correlation coefficients, should be distinguished from biological redundancy, which refers to overlap in the physiological constructs represented by predictors. Because both PNI and GNRI incorporate serum albumin and reflect related aspects of nutritional status, their independent hazard ratios may be difficult to interpret when entered into the same model. Thus, the concern is that mutually adjusted hazard ratios for two albumin-based indices may not clearly represent the overall prognostic value of either index.

A more appropriate strategy would be to evaluate PNI and GNRI in separate multivariable models and compare their prognostic performance against the same baseline clinical model. If available, reporting the observed correlation between PNI and GNRI, together with VIF and tolerance values, would further help readers judge whether the two indices provide complementary or redundant prognostic information. Model performance could then be compared using Harrell's C-index, time-dependent AUC, calibration, and, where appropriate, decision-curve analysis. In the presence of competing risks, discrimination measures specifically adapted for competing-risk prediction should also be considered.

In summary, this study provides clinically relevant evidence supporting the prognostic importance of nutritional status in elderly patients with early-stage prostate cancer. To strengthen the methodological rigor and practical utility of the findings, we suggest that the authors consider the following supplementary analyses if the relevant data are available: first, classify deaths as prostate cancer-specific or competing non-cancer deaths and estimate cumulative incidence functions; second, apply a Fine–Gray subdistribution hazard model using standard software such as the cmprsk package in R or stcrreg in Stata; third, report the correlation, VIF, and tolerance values for PNI and GNRI; and fourth, compare separate PNI- and GNRI-based models using Harrell's C-index, AUC, calibration, and competing-risk-adapted discrimination metrics. These analyses would clarify whether PNI and GNRI are independent prognostic markers, alternative nutritional risk tools, or overlapping indicators of systemic vulnerability in elderly patients with early-stage prostate cancer.

## References

[1] Peker P Geçgel A Özkan O Yilmaz S Selvi S Cirik C . Impact of prognostic nutritional index and geriatric nutritional risk index on prognosis in elderly patients with early-stage prostate cancer. Front Nutr. (2026) 13:1745718. doi: 10.3389/fnut.2026.1745718 41783818 PMC12953100

[2] Boyle HJ Alibhai S Decoster L Efstathiou E Fizazi K Mottet N . Updated recommendations of the International Society of Geriatric Oncology on prostate cancer management in older patients. Eur J Cancer. (2019) 116:116–36. doi: 10.1016/j.ejca.2019.04.031 31195356

[3] Daskivich TJ Fan KH Koyama T Albertsen PC Goodman M Hamilton AS . Effect of age, tumor risk, and comorbidity on competing risks for survival in a US population-based cohort of men with prostate cancer. Ann Intern Med. (2013) 158:709–17. doi: 10.7326/0003-4819-158-10-201305210-00005 23689764 PMC3760479

[4] Yan D Shen Z Zhang S Hu L Sun Q Xu K . Prognostic values of geriatric nutritional risk index (GNRI) and prognostic nutritional index (PNI) in elderly patients with Diffuse Large B-Cell Lymphoma. J Cancer. (2021) 12:7010–7. doi: 10.7150/jca.62340 34729103 PMC8558670

[5] Albertsen PC Moore DF Shih W Lin Y Li H Lu-Yao GL. Impact of comorbidity on survival among men with localized prostate cancer. J Clin Oncol. (2011) 29:1335–41. doi: 10.1200/JCO.2010.31.2330 21357791 PMC3084001

